# Supplementary figures and images for: High Expression of the Lysosomal Protease Cathepsin D Confers Better Prognosis in Neuroblastoma Patients by Contrasting EGF-Induced Neuroblastoma Cell Growth
Source: Int J Mol Sci. 2022 Apr 26;23(9):4782. doi: 10.3390/ijms23094782 (PMC9101173; doi:10.3390/ijms23094782)

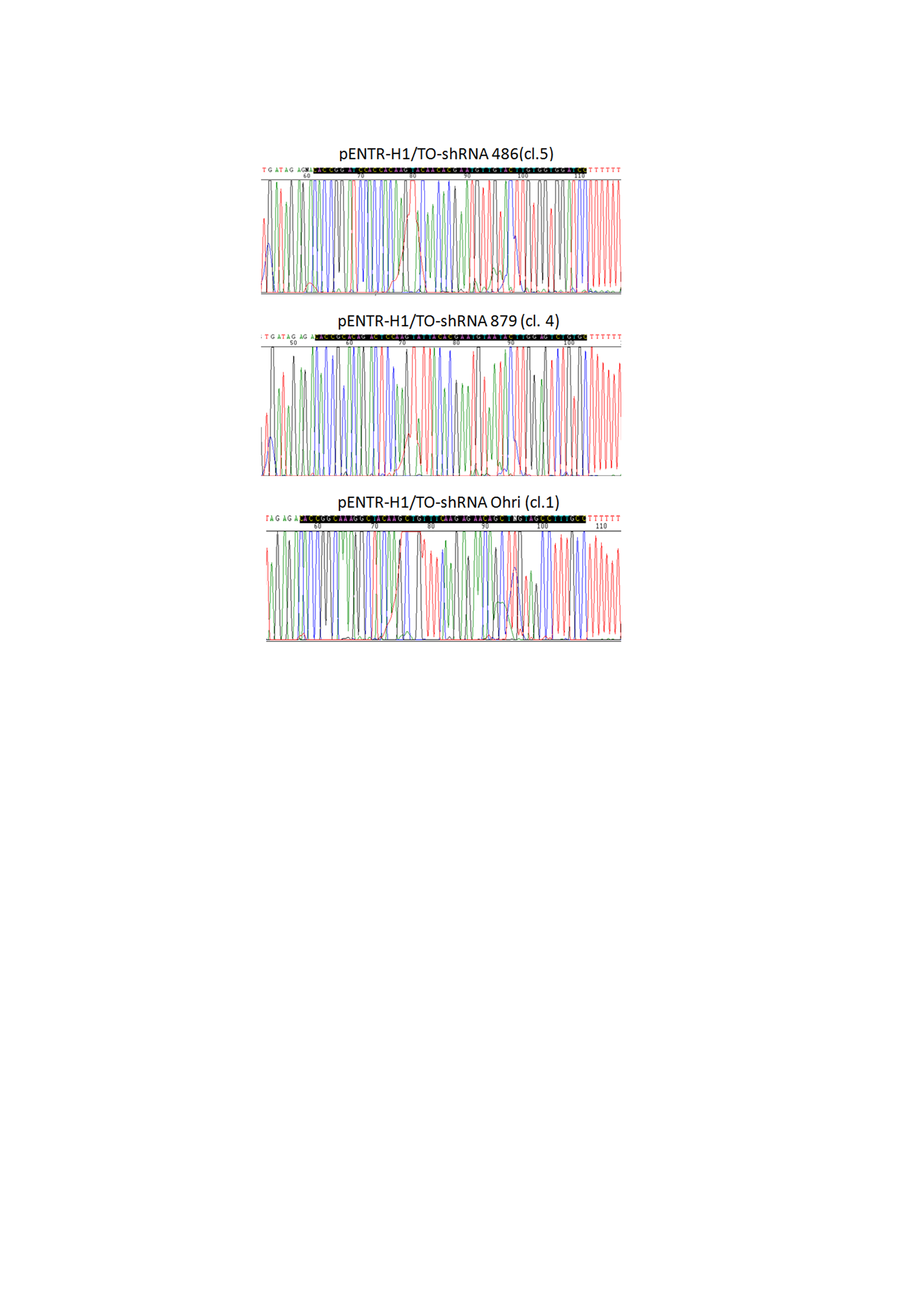

Supplement: Supplementary file 1 [file ijms-23-04782-s001.zip › ijms-1692054-supplementary.tif]
